# Retraction: MiR-203 Suppresses ZNF217 Upregulation in Colorectal Cancer and Its Oncogenicity

**DOI:** 10.1371/journal.pone.0244268

**Published:** 2020-12-17

**Authors:** The PLOS ONE Editors

Following the publication of this article [1], concerns were raised regarding the results presented in Figs 2, 4 and 5, as well as similarities between panels presented in this article and an article previously published in *PLOS ONE* [2].

Specifically,

- The following panels appear similar:
  ○ The Invasion NC siRNA panel of Fig 2C [1] and the Migration siRNA NC panel of Fig 5C [1].
  ○ The Migration NC panel of Fig 4C [1], the Migration NC panel of Fig 4D [1], and the SW480 miR-210 mimics panel of Fig 4A [2].
  ○ The Migration miR-203 mimics panel of Fig 4C [1], the Migration miR-203 inhibitor panel of Fig 4D [1], and the SW480 miR-210 inhibitor panel of Fig 4B [2].
  ○ The Invasion NC panel of Fig 4C [1], the Invasion NC panel of Fig 4D [1], and the SW480 miR-210 mimics panel of Fig 4C [2].
  ○ The Invasion miR-203 mimics panel of Fig 4C [1], the Invasion miR-203 inhibitor panel of Fig 4D [1], and the SW480 miR-210 inhibitor panel of Fig 4D [2].
  ○ The Migration miR203 mimics panel of Fig 5C [1] and the Migration Normoxia-NC panel of Fig 5D [2].
  ○ The Migration siRNA ZNF217 panel of Fig 5C [1] and the Migration Hypoxia-NC panel of Fig 5B [2].
  ○ The Invasion miR203 mimics panel of Fig 5C [1] and the SW480 NC panel of Fig 4C [2].
  ○ The Invasion siRNA NC panel of Fig 5C [1] and the Migration Hypoxia-mimics panel of Fig 5B [2].
- Partial overlap has been detected between the following panels:
  ○ The Invasion ZNF217 siRNA panel of Fig 2C [1] and the Migration Hypoxia-inhibitor panel of Fig 5D [2].
  ○ The Invasion NC panel of Fig 4C [1], the Invasion NC panel of Fig 4D [1], and the SW480 miR-210 mimics panel of Fig 4C [2] partially overlap with the following panels:
    ■ Invasion miR203 mimics panel of Fig 5C [1].
    ■ Invasion siRNA ZNF217 of Fig 5C [1].
    ■ SW480 NC of Fig 4C [2].
    ■ SW480 NC of Fig 4D [2].
    ■ Invasion Normoxia-NC of Fig 5B [2].
    ■ Invasion Hypoxia-inhibitor of Fig 5D [2].

The authors stated that the similarity observed between the panels described above are the result of inadvertent errors introduced during figure preparation and provided data underlying their published results. However, the extent of the image concerns observed in this article raise serious concerns regarding the handling of the data obtained during this study and the integrity of the published results. In light of the concerns affecting multiple figure panels that question the integrity of these data, the *PLOS ONE* Editors retract this article.

XZ and CW agreed with the retraction. ZL, LD, ZD, YY, LW, JL, GZ, and AQ either did not respond directly or could not be reached.
